# Supplementary material for: Thermal acclimation mitigates cold-induced paracellular leak from the Drosophila gut
Source: Sci Rep. 2017 Aug 18;7:8807. doi: 10.1038/s41598-017-08926-7 (PMC5562827; doi:10.1038/s41598-017-08926-7)
Supplement: Supplementary file 1 — Supplementary Information [file 41598_2017_8926_MOESM1_ESM.pdf]

SUPPLEMENTARY FIGURES AND TABLES

Thermal acclimation mitigates cold-induced paracellular leak from the *Drosophila* gut

Heath A. MacMillan*, Gil Yerushalmi, Sima Jonusaite, Scott P. Kelly and Andrew Donini

Correspondence to: heath.macmillan@carleton.ca

Table S1. Changes in mRNA abundance of known or putative septate junction genes (those included in the Gene Ontology record "Septate Junction Assembly" for *Drosophila melanogaster*) in response to thermal acclimation. Adult male flies were acclimated to 6°C (cold) or 21.5°C (warm) and snap frozen for RNAseq analysis. Data from MacMillan et al. (MacMillan et al., 2016) – see referenced study for further details. Genes in bold were significantly differentially expressed in response to thermal acclimation following false discovery rate correction (*Q*-value). Blue = higher in cold-acclimated flies (20/34 genes, 59%), Red = higher in warm-acclimated flies (2/34 genes, 6%). Genes marked with an X were further investigated in the present study.

Gene name	Symbol	Flybase ID	Fold change	*P*-value	*Q*-value	This study
Na pump α subunit	Atpα	FBgn0002921	0.84	0.078	0.166	
**big bang**	**bbg**	**FBgn0087007**	**0.75**	**0.003**	**0.009**	
boudin	bou	FBgn0261284	0.96	0.891	0.943	
**coiled**	**cold**	**FBgn0031268**	**1.31**	**0.007**	**0.020**	
Contactin	Cont	FBgn0037240	1.18	0.074	0.159	
coracle	cora	FBgn0010434	0.93	0.313	0.481	**X**
crimpled	crim	FBgn0036198	0.99	0.956	0.976	
**crooked**	**crok**	**FBgn0032421**	**1.79**	**<0.0001**	**<0.0001**	
**discs large**	**dlg1**	**FBgn0001624**	**1.35**	**0.004**	**0.014**	**X**
G protein α i subunit	Gαi	FBgn0001104	1.15	0.089	0.186	
**G protein α o subunit**	**Gαo**	**FBgn0001122**	**1.71**	**<0.0001**	**0.0002**	
**Germinal centre kinase III**	**GckIII**	**FBgn0266465**	**0.81**	**0.0084**	**0.025**	
**gliotactin**	**Gli**	**FBgn0001987**	**1.65**	**<0.0001**	**<0.0001**	
**kune-kune**	**kune**	**FBgn0033032**	**1.22**	**0.015**	**0.041**	**X**
**lethal (2) giant larvae**	**l(2)gl**	**FBgn0002121**	**1.74**	**<0.0001**	**<0.0001**	
**lachesin**	**Lac**	**FBgn0010238**	**1.98**	**<0.0001**	**<0.0001**	
locomotion defects	loco	FBgn0020278	1.08	0.548	0.708	
**Macroglobulin complement-related**	**Mcr**	**FBgn0267488**	**1.29**	**0.0004**	**0.002**	
**mesh**	**mesh**	**FBgn0051004**	**0.68**	**<0.0001**	**<0.0001**	**X**
moody	moody	FBgn0025631	0.97	0.674	0.806	
**neuroglian**	**Nrg**	**FBgn0264975**	**1.63**	**<0.0001**	**<0.0001**	
**nrv2**	**nrv2**	**FBgn0015777**	**1.94**	**<0.0001**	**<0.0001**	
**neurexin IV**	**Nrx-IV**	**FBgn0013997**	**1.69**	**<0.0001**	**<0.0001**	
p21-activated kinase	Pak	FBgn0267698	1.06	0.557	0.716	
pasiflora 1	pasi1	FBgn0038545	1.35	0.051	0.118	
**pasiflora 2**	**pasi2**	**FBgn0037680**	**1.59**	**<0.0001**	**0.0002**	
**pickle**	**pck**	**FBgn0013720**	**1.33**	**0.002**	**0.006**	
scribbled	scrib	FBgn0263289	0.78	0.521	0.685	**X**
**sinuous**	**sinu**	**FBgn0010894**	**1.45**	**<0.0001**	**<0.0001**	
**snakeskin**	**Ssk**	**FBgn0036945**	**1.38**	**0.015**	**0.041**	
**melanotransferin**	**Tsf2**	**FBgn0036299**	**1.48**	**<0.0001**	**<0.0001**	
**varicose**	**vari**	**FBgn0250785**	**1.50**	**<0.0001**	**<0.0001**	

Table S2. Antibodies used for immunoblotting in the present study (marked with an X in Table S1). DSHB: Developmental Studies Hybridoma Bank (Iowa City, IA, USA); SCB: Santa Cruz Biotechnology (Mississauga, ON, Canada).

**Protein (*gene*)**	**Antibody source**	**Antibody catalog number or citation or epitope sequence**	**Antibody dilution factor**	**Secondary antibody host**
Coracle (*cora*)	DSHB	C566.9	1:250	Mouse
Discs large (*dlg1*)	DSHB	4F3	1:400	Mouse
Scribble (*scrib*)	SCB	sc-26941	1:250	Goat
Mesh (*mesh*)	Prof. Mikio Furuse	(Izumi et al., 2012)	1:2000	Rabbit
Kune-kune (*kune*)	Prof. Mikio Furuse	(Nelson et al., 2010)	1:1000	Rabbit

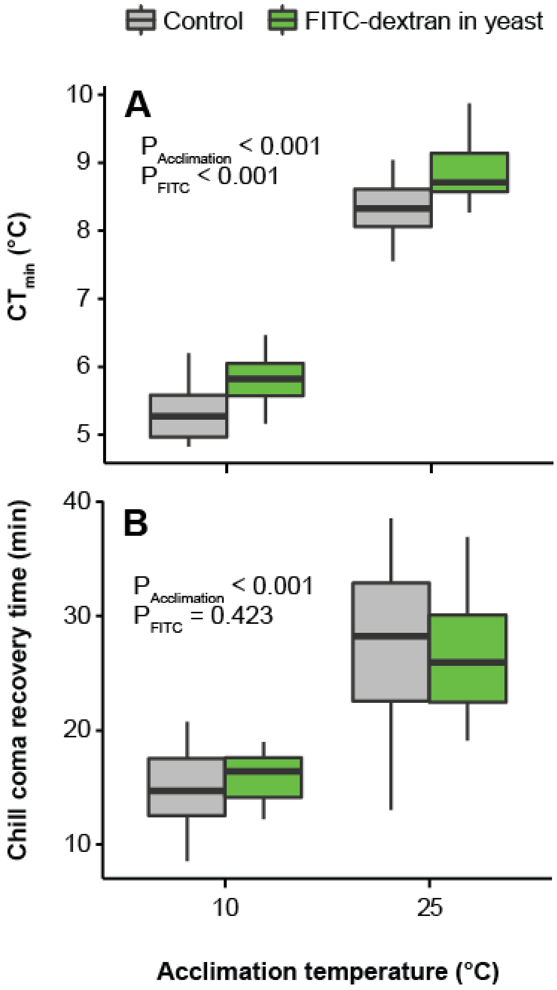

Figure S1. The effects of feeding on FITC-dextran in yeast on cold tolerance of *D. melanogaster* females. Critical thermal minimum (CT_{min}; A) and chill coma recovery time (CCRT; B) were measured on flies fed for 24h on a 50 µL droplet of a 2.5% (w/v) solution of FITC-dextran in water, mixed with 10 mg of active brewers yeast. Feeding on the FITC-dextran yeast mixture slightly increased the CT_{min}, and had no effect on CCRT. In the case of CT_{min}, the effects of cold acclimation remained very similar regardless of an overall increase in the CT_{min} as an effect of feeding.

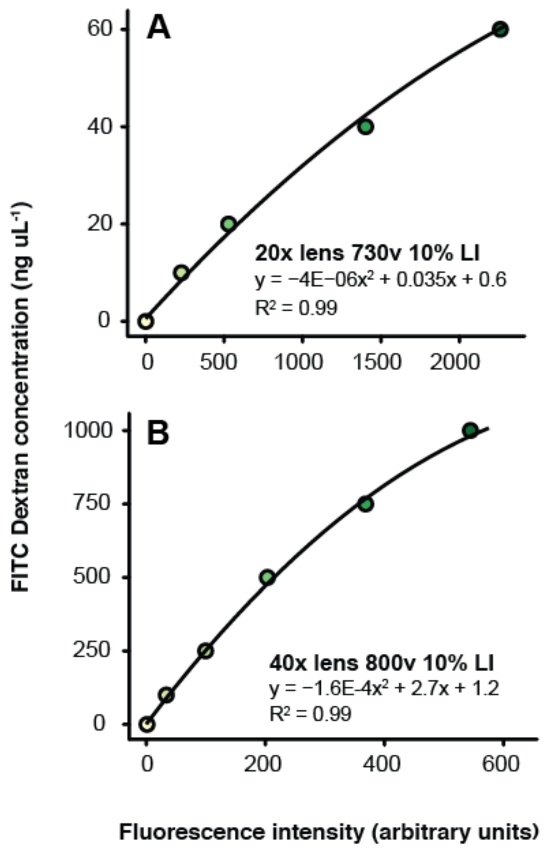

Figure S2. Standard curves of FITC-dextran concentration measured by confocal microscopy of solutions inside rectangular microcapillaries. FITC-dextran was quantified in microcapillaries as previously described for primary urine samples from crickets and *Drosophila* by Leader and O'Donnell (Leader and O'Donnell, 2005). Two different groups of settings were used to quantify FITC-dextran concentrations in the hemolymph to ensure accurate estimates across a broad concentration range. Points represent means of n=3 replicates at each concentration. A) Standard curve using 20x lens for lower concentrations B) Standard curve using 40x lens for higher concentrations.

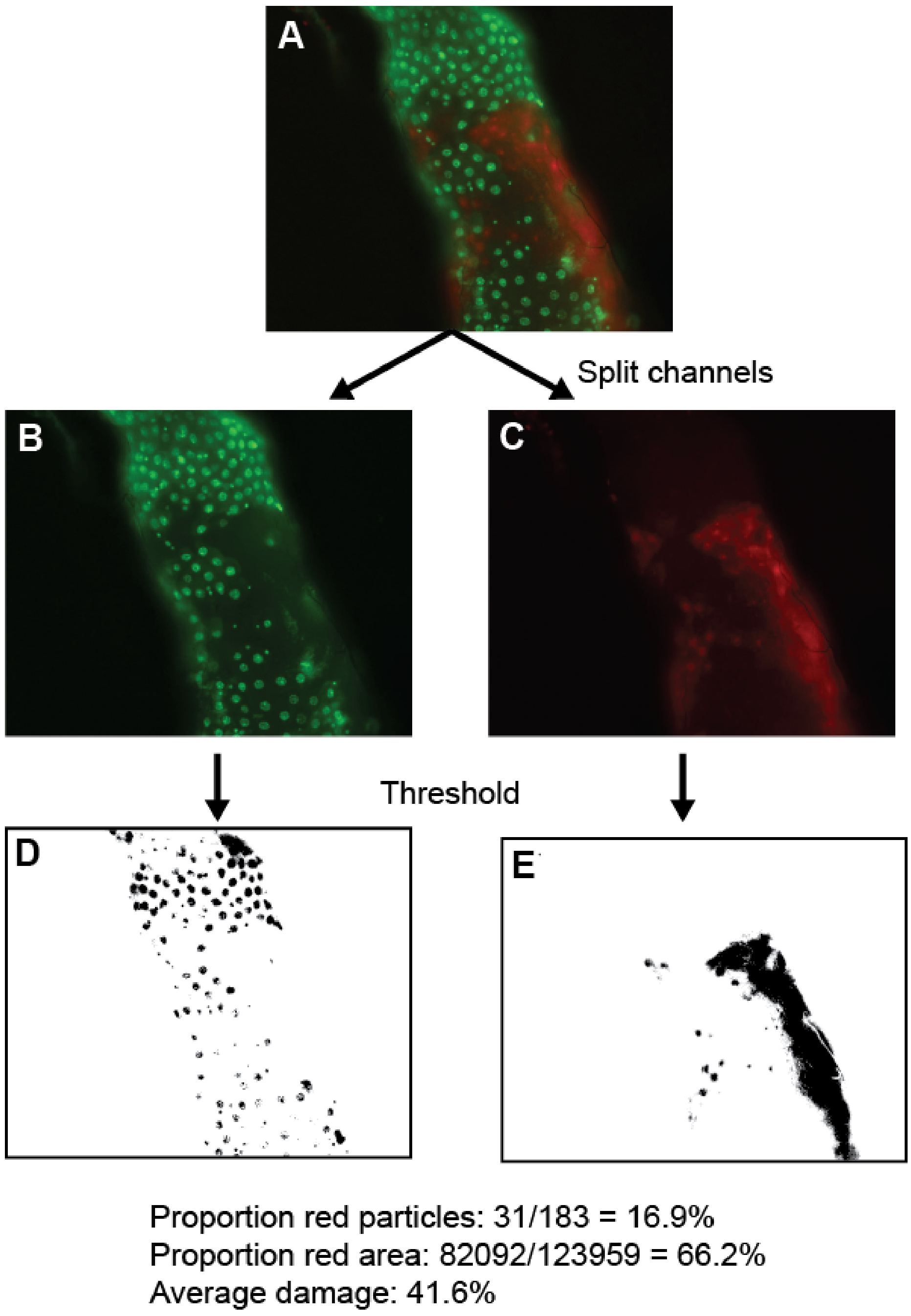

Figure S3. Tissue damage image analysis method in FIJI. Composite images (A) were split into their component green (live cells, B) and red (damaged cells, C) channels. Each channel was adjusted by automated thresholding in FIJI to produce black and white images representing areas of live or dead cells. The number of particles and proportion of area that stained as green and red were each calculated and used to estimate a proportion of the tissue that was damaged. Both Sybr-14 and propridium iodide bind DNA, but propridium iodide can only enter the cytoplasm of cells that have a damaged cell membrane. Because DNA is degraded during cell death, the two dyes produce different staining patterns. For this reason, the two methods of quantifying red staining relative to green staining are averaged to produce a final estimate of tissue damage.

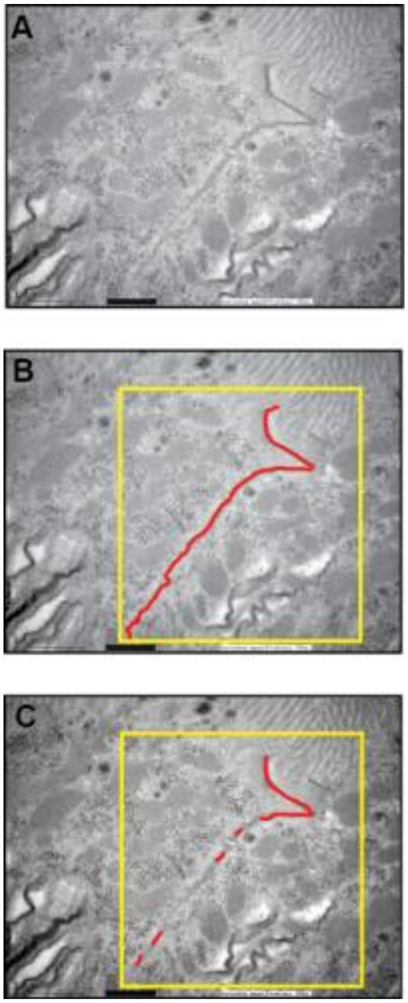

Figure S4. Method for quantification of the cell-cell contact region and septate junctions in sections of the posterior midgut of female *Drosophila melanogaster*. Lengths of cell-cell contact and septate junctions were quantified by an author blind to the identity of the images. (A) Original image, (B) tracing of path of cell-cell contact, and (C) tracing of identified septate junctions. A 750 × 750 pixel region of the image was outlined around the region of cell-cell contact (yellow square) and within that region the path of cell-cell contact was digitally traced. Septate junctions were identified as dark regions where the two cell membranes lay in close parallel contact. Select septa were verified using higher magnification images.
